# Evaluating geographical disparities on clinical outcomes following cytoreductive surgery and hyperthermic intraperitoneal chemotherapy

**DOI:** 10.1007/s10151-024-02911-9

**Published:** 2024-02-20

**Authors:** Adrian Siu, Daniel Steffens, Nabila Ansari, Sascha Karunaratne, Henna Solanki, Nima Ahmadi, Michael Solomon, Brendan Moran, Cherry Koh

**Affiliations:** 1 Surgical Outcomes Research Centre (SOuRCe), Royal Prince Alfred Hospital, C/O Royal Prince Alfred Hospital, Missenden Road, PO Box M 157, Camperdown, NSW 2050 Australia; 2 Faculty of Medicine and Health, Central Clinical School, The University of Sydney, Sydney, NSW Australia; 3 Department of Colorectal Surgery, Royal Prince Alfred Hospital, Sydney, NSW Australia; 4 Peritoneal Malignancy Institute, North Hampshire Foundation Trust, Basingstoke, UK

**Keywords:** Cytoreductive surgery, Hyperthermic intraperitoneal chemotherapy, Peritoneal malignancy, Surgical outcomes, Oncological outcomes

## Abstract

**Background:**

Rural Australians typically encounter disparities in healthcare access leading to adverse health outcomes, delayed diagnosis and reduced quality of life (QoL) parameters. These disparities may be exacerbated in advanced malignancies, where treatment is only available at highly specialised centres with appropriate multidisciplinary expertise. Thus, this study aims to determine the association between patient residence on oncological, surgical and QoL outcomes following cytoreductive surgery (CRS) and hyperthermic intra-peritoneal chemotherapy (HIPEC).

**Methods:**

A retrospective analysis was conducted on consecutive patients undergoing CRS and HIPEC at Royal Prince Alfred Hospital from January 2017 to March 2022. On the basis of their postcode of residence, patients were stratified into metropolitan and regional groups. Data encompassing demographics, oncological, surgical and QoL outcomes were compared. Statistical analysis included chi-square test, *t*-tests and Kaplan–Meier survival curves.

**Results:**

Among the 317 patients, 228 (72%) were categorised as metropolitan and 89 (28%) as regional. Metropolitan patients presented higher rates of recurrence (61.8% versus 40.0%, *p* = 0.014) and shorter overall mean survival [3.8 years (95% CI: 3.44–4.09) versus 4.2 years (95% CI: 3.76–4.63), *p* = 0.019] compared with regional patients. No other statistically significant differences were observed in oncological, surgical and QoL outcomes.

**Conclusions:**

Most oncological, surgical and QoL parameters did not differ by geographical location of patients undergoing CRS and HIPEC for peritoneal malignancies at a high-volume quaternary referral centre. Observed differences in recurrence and survival may be attributed to the selective nature of surgical referrals and variable follow-up patterns. Future research should focus on characterising referral pathways and its influence on post-operative outcomes.

## Introduction

Collectively, the Australian population enjoys the advantages of a robust and affordable healthcare system, leading to higher life expectancies and favourable populational health when compared with similarly developed nations [1]. However, for approximately 30% of Australians residing in regional locations [2], their healthcare experiences and health outcomes differ from those in metropolitan areas. Unfortunately, owing to the unique geography of Australia, regional residents often need to travel large distances to access their nearest primary or secondary healthcare facility. As a consequence of this, rural communities often experience an amplification of multiple indicators of health disparities, and studies have demonstrated reduced quality of life (QoL), elevated rates of mortality [3–5] and worse median overall survival (94 versus 104 months, *p* < 0.001) for colon cancer outcomes when compared with urban communities [6].

Despite experiencing poorer health outcomes and reduced life expectancy in regional Australia, these patients often face limited access to healthcare services, with studies revealing significantly longer delays in accessing specialist care [7, 8]. To address this healthcare disparity, innovative care models have been trialed, including visiting medical specialists, expansion of telehealth and virtual capabilities and increasing health infrastructure [9, 10]. However, as these innovations continue to evolve, review studies have highlighted the multifaceted nature of rural healthcare provision, with major challenges related to ethnicity, socioeconomic status and inadequate communication within the health system [11, 12]. Additionally, while these innovative models may suffice for acute pathologies, some complex conditions require a multidisciplinary approach and highly specialised care. In these situations, it often becomes necessary for the patient to travel to quaternary centres to receive this level of care. An example of this specialised and complex pathology is the management of peritoneal malignancy.

The majority of peritoneal malignancies occur as a result of trans-coelomic metastasis from an advanced primary cancer into the peritoneal cavity. Most frequently, this involves tumours of the appendix, stomach, colon, ovaries, pancreas or gallbladder [13]. To manage peritoneal malignancy, the optimal therapeutic approach often includes adopting multimodal therapy, encompassing surgical intervention, chemotherapy and targeted therapy, which has shown a survival benefit when compared with traditional palliative approaches (60 months versus 4–12 months) [14, 15]. In the standard procedure, these patients undergo cytoreductive surgery (CRS) and hyperthermic intraperitoneal chemotherapy (HIPEC). The former involves multiple peritonectomy procedures and visceral resections to remove all macroscopic disease and the latter for the elimination of microscopic disease [16]. Although CRS and HIPEC are considered the gold-standard for managing colorectal peritoneal malignancy, ongoing debate and criticism continues regarding the potential of this strategy resulting in high morbidity and mortality [16, 17].

To ensure maximal benefit from CRS and HIPEC, early diagnosis of peritoneal malignancy is imperative and requires a multidisciplinary team that includes surgeons, medical oncologists, anaesthesiologists, intensivists, radiologists and pathologists [16]. However, the challenges of geographic remoteness, complex socio-cultural barriers, limited experience and varying levels of healthcare infrastructure [18] may negatively impact the early diagnosis of peritoneal malignancy in regional settings. While there continues to be research on reducing healthcare disparities in these communities, there is limited data regarding the outcomes of patients who travel considerable distances to access this highly specialised treatment.

Therefore, the purpose of this study is to explore the influence of patient residence on post-operative outcomes after CRS and HIPEC for peritoneal malignancy. The primary outcomes were oncological parameters [peritoneal carcinomatosis index (PCI) and completeness of cytoreduction (CC)], surgical outcomes [length of stay (LOS), complications and overall survival] and QoL measurements.

## Methods

### Study design and setting

A retrospective cohort study of patients who underwent CRS and HIPEC at Royal Prince Alfred Hospital, Sydney, Australia, was conducted from April 2017 to March 2022. Recommended reporting guidelines from the Strengthening the Reporting of Observational Studies in Epidemiology (STROBE) guidelines were followed [19]. Relevant ethical and governance approval were obtained from the Sydney Local Health District Human Ethics Review Committee (2019/ETH07574).

All patient demographics, oncological, surgical and recurrence data originated from the PREMIER (Peritonectomy Surgical Research Program) database, which routinely collects information on oncological parameters and surgical outcomes. A waiver of ethical consent was approved to access this de-identified data. For the QoL measurements of this study, consecutive patients were invited to participate by their surgeon during their assessment for CRS and HIPEC. Only patients with baseline questionnaire responses and informed and written consent were included in the study.

### Participants

The inclusion criteria consisted of patients who were planning to undergo CRS and HIPEC for peritoneal malignancy, able to provide informed consent, and had the ability to complete a self-reported questionnaire either independently or with the assistance of relatives/caregiver. The exclusion criteria were patients who declined participation in the study, were missing baseline questionnaire responses, or resided in inter-state locations outside of New South Wales, Australia.

### Outcomes

The Australia Statistical Geography Standard-Remoteness Area (ASGS-RA) classification was used to stratify patients’ postcode of residence into “metropolitan”, “regional” and “remote” [20]. This measure of remoteness is based on road distance from populated locations to five categories of service centres and uses population as a proxy for measuring availability of services. Patients who resided in a “major city” according to ASGS-RA were classified as “metropolitan”, while patients who resided in “inner regional”, “outer regional”, “remote” and “very remote” were classified as “regional”.

The oncological parameters were PCI and CC scores. The PCI is a standardised score used in peritoneal metastases that quantifies the extent of disease in 13 different abdominopelvic regions and ranges from 0 to 39 [21, 22]. Intra-operatively, a score of “0” is designated as no disease, “1” as disease up to 0.5 cm, “2” as disease up to 5 cm and “3” as disease that is a confluence of unresectable disease or > 5 cm. The CC score is a prognostic indicator and categorises the residual disease at the end of cytoreductive surgery, ranging from CC-0–CC-3. A score of CC-0 indicates no residual disease after CRS, CC-1 indicates residual tumour nodules (< 2.5 mm), CC-2 indicates residual tumour nodules between 2.5 mm and 2.5 cm and CC-3 for persisting tumour nodules > 2.5 cm or confluence of unresectable disease [23]. For all pathologies [excluding pseudomyxoma peritonei (PMP)], the CC score was stratified into CC = 0 and CC > 0. However, owing to the phenotypical and biological differences of PMP and other peritoneal pathologies, the CC score was re-stratified into CC ≤ 1 and CC ≥ 2 [24].

The surgical outcomes included blood loss (mL), blood transfusion status, post-operative complications, length of stay in the intensive care unit (ICU), post-operative length of stay (LOS) and severity of complications (using Clavien–Dindo classification [25]). The principle of Clavien–Dindo classification is based on the therapy needed to treat the complication and ranges from grade I (any deviation from the post-operative course) to grade V (death of a patient) [26]. The severity of complications was dichotomised into minor (grade I–II) or major (grade III–V). Survival data was captured from the Australian Registry of Births, Deaths & Marriage. The overall survival was calculated in years from the date of surgery till death or last time of recorded contact with the patient, censured in June 2023. The duration of follow-up was taken from the date of surgery to June 2023.

The QoL data was measured using the 36-item Short-Form Survey version 2 (SF-36v2) [27]. The SF-36v2 tool is a reliable and valid tool, which has been extensively used in patients undergoing CRS and HIPEC [28, 29]. This QoL data was collected longitudinally at various time points: pre-operatively, prior to discharge and 3, 6 and 12 months after surgery. The SF-36v2 produces two summary health scores: the physical component score (PCS), which is comprised of the domains physical functioning, role physical, bodily pain and general health, and the mental component score (MCS), which includes the domains vitality, social functioning, role – emotional and mental health. The non-standardised range of each domain is 0–100, with 0 being the lowest QOL for that domain and 100 the highest QOL. These scores are normalised to the general population through linear transformation [with a mean score of 50, standard deviation (SD) 10] in accordance with the SF-36 scoring manual [30]. A higher standardised score indicates better QOL parameters.

### Statistical analyses

Statistical analysis was performed using IBM SPSS® Statistics version 29. Descriptive analyses were undertaken, and all parameters were reported separately for three groups of patients (overall, metropolitan and regional). Categorical variables were reported in frequency (percentage), and continuous variables were summarised using either mean and SD or median and interquartile range (IQR). Difference between metropolitan and regional groups for demographics, oncological, surgical and QoL parameters were evaluated using chi-square test for categorical variables and *t*-test for continuous variables. Kaplan–Meier survival curves were used to estimate overall survival, with differences between groups assessed by the log rank test. All statistical tests considered a *p*-value of < 0.05 to be significant.

## Results

### Patient characteristics

A total of 351 patients underwent CRS and HIPEC from April 2017 to March 2022. Of these, 15 patients had no baseline QoL data, and 19 patients were excluded, as they listed an inter-state postcode of residence. The final included cohort consisted of 317 patients (90%), and the majority (72%, *n* =  28) were classified as metropolitan. Overall, the mean age was 54.4 (SD: 13.7) years, and 138 patients were male (43.5%). The primary tumour pathology was colorectal cancer (44.8%, *n* = 142), followed by appendix adenocarcinoma (22.1%, *n* = 70), pseudomyxoma peritonei (16.7%, *n* = 53) and other peritoneal malignancies [ovarian (6.6%, *n* = 21), small bowel adenocarcinoma (1.9%, *n* = 6) and peritoneal mesothelioma (5.7%, *n* = 18)]. The primary discharge destination for the cohort was home (90.5%, *n* = 287), and 23/317 (7.3%) required re-admission into a hospital following discharge (Table 1).

**Table 1:** Patient characteristics (*n* = 317)

Variables	Overall (*n* = 317)	Metropolitan (*n* = 228)	Regional (*n* = 89)	*p*-Value
Age, years	54.4 ± 13.7	54.0 ± 13.3	55.5 ± 14.8	0.368
Sex				0.115
Female	179 (56.5%)	135 (59.2%)	44 (49.4%)	
Male	138 (43.5%)	93 (40.8%)	45 (50.6%)	
Pathology				0.286
Pseudomyxoma peritonei	53 (16.7%)	39 (17.1%)	14 (15.7%)	
Appendix adenocarcinoma	70 (22.1%)	46 (20.2%)	24 (27.0%)	
Colorectal	142 (44.8%)	103 (45.2%)	39 (43.8%)	
Ovarian	21 (6.6%)	19 (8.3%)	2 (2.2%)	
Peritoneal mesothelioma	18 (5.7%)	11 (4.8%)	7 (7.9%)	
Other**	13 (4.1%)	10 (4.4%)	3 (3.4%)	
Discharge destination				0.501
Home	287 (90.5%)	208 (91.2%)	79 (88.8%)	
Rehabilitation/other hospital	30 (9.5%)	20 (8.8%)	10 (11.2%)	
30-day re-admission				0.088
Yes	23 (7.3%)	13 (5.7%)	10 (11.2%)	
No	294 (92.7%)	215 (94.3%)	79 (88.8%)	

Data presented as mean ± standard deviation or frequency (percentage)

*Sample < 317 indicates missing data

**Small bowel adenocarcinoma (*n* = 6), primary peritoneal (*n* = 1) and other pathologies (*n* = 6)

Completion rates for the QoL questionnaire were 75.4% (239/317) pre-operatively, 74.0% (233/315) prior to discharge, 63.0% (194/308) at 3 months, 61.4% (178/290) at 6 months and 51.3% (118/230) at 12 months. For all patients, the mean follow-up time was 2.46 years (SD: 30.3 months). Missing QoL data were not imputed.

### Oncological parameters

The median PCI was similar in the metropolitan group compared with regional group (12 versus 13, *p* = 0.484). When comparing PCI severity, there was a higher proportion of metropolitan patients with PCI < 15 compared with regional patients; however, this was not statistically significant (56.6% versus 52.8%, *p* = 0.544). Excluding PMP patients, a score of CC-0 was achieved in the majority of metropolitan (84.1%, *n* = 159) and regional patients (80.0%, *n* = 60). In PMP patients only, a clearance score of CC-0 or CC-1 was achieved in the majority of metropolitan patients (87.2%, *n* = 34) and all regional patients (100%, *n* = 14, *p* = 0.309; Table 2). No statistical differences were observed between metropolitan and regional patients for all oncological parameters.

**Table 2:** Oncological parameters between metropolitan and regional patients (*n* = 317)

Variables	Overall (*n* = 317)	Metropolitan (*n* = 228)	Regional (*n* = 89)	*p*-Value
Neoadjuvant chemotherapy				0.230
Yes	17 (5.4%)	15 (6.6%)	2 (2.3%)	
No	92 (29.0%)	65 (28.5%)	27 (30.3%)	
Missing	208 (65.6%)	148 (64.9%)	60 (67.4%)	
PCI score	12 (6–24)	12 (6–23)	13 (7–13)	0.484
PCI severity				0.544
PCI < 15	176 (55.5%)	129 (56.6%)	47 (52.8%)	
PCI ${\text{f}}$ 15	141 (44.5%)	99 (43.4%)	42 (47.2%)	
CC score (excluding PMP)				0.469
CC = 0	219 (83.0%)	159 (84.1%)	60 (80.0%)	
CC > 0	45 (17.0%)	30 (15.9%)	15 (20.0%)	
CC score (only PMP)				0.309
CC ≤ 1	48 (90.6%)	34 (87.2%)	14 (100.0%)	
CC ≥ 2	5 (9.4%)	5 (12.8%)	–	

Data presented as median (interquartile range) or frequency (percentage). *PCI* peritoneal cancer index, *CC* completeness of cytoreduction, *PMP* pseudomyxoma peritonei

*Sample < 317 indicates missing data

### Surgical outcomes

The mean blood loss for metropolitan and regional patients was 1548.0 and 1561.4 mL, respectively (*p* = 0.955), and a similar proportion required blood transfusion (52.2% versus 59.1%, *p* = 0.270). Post-operatively, most metropolitan (76.1%, *n* = 172) and regional patients (77.3%, *n* = 68) experienced at least one post-operative complication; however, most of these were considered minor (grade I–II) in both groups (65.5% and 59.4%, respectively) [25]. The mean ICU LOS was similar for metropolitan and regional groups (5.3 and 5.2 days, respectively). Comparing groups, metropolitan patients recorded a mean post-operative LOS of 21.0 days (SD: 16.7) and regional patients 20.1 days (SD: 10.8, *p* = 0.619; Table 3).

**Table 3:** Surgical outcomes between metropolitan and regional patients (*n* = 317)

Variables	Overall (*n* = 317)	Metropolitan (*n* = 228)	Regional (*n* = 89)	*p*-Value
Blood loss, mL*	1551.8 ± 1854.9	1548.0 ± 2030.0	1561.4 ± 1316.2	0.955
Blood transfusion*				0.270
Yes	171 (54.1%)	119 (52.2%)	52 (59.1%)	
No	145 (45.9%)	109 (47.8%)	36 (40.9%)	
Post-operative complication*				0.827
Yes	240 (76.4%)	172 (76.1%)	68 (77.3%)	
No	74 (23.6%)	54 (23.9%)	20 (22.7%)	
Severity of complication*				0.376
Grade I–II	153 (63.8%)	112 (65.5%)	41 (59.4%)	
Grade III–V	87 (36.2%)	59 (34.5%)	28 (40.6%)	
ICU LOS, days	5.3 ± 3.3	5.3 ± 3.3	5.2 ± 3.3	0.749
Post-operative LOS, days*	20.8 ± 15.3	21.0 ± 16.7	20.1 ± 10.8	0.619

Data presented as mean ± standard deviation or frequency (percentage), *LOS* length of stay

*Sample < 317 indicates missing data

## Survival and recurrence

**Fig. 1:**
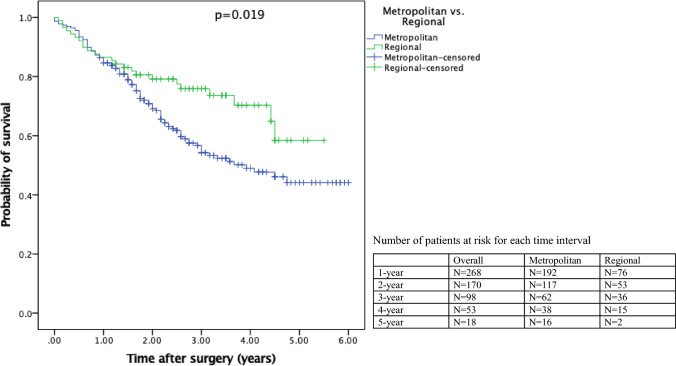
Kaplan–Meier analysis of the overall survival after CRS and HIPEC, where *n* = 228 for metropolitan and *n* = 89 for regional (*n* = 317)

Mean overall survival was worse in the metropolitan group, at 3.77 years (95% CI: 3.44–4.09) compared with the regional group, at 4.20 years (95% CI: 3.76–4.63, *p* = 0.019; Fig. 1). Further comparison of the overall survival rate found that the metropolitan group was worse compared with regional group for 1-year (84.6% versus 85.9%), 2-year (69.1% versus 79.2%), 3-year (54.2% versus 75.9%) and 5-year survival (44.1% versus 58.4%). For recurrence, metropolitan patients had higher rates compared with regional patients (61.8% versus 40.0%, *p* = 0.012; Table 4).

**Table 4:** Overall survival rates and recurrence

	Overall (*n* = 317)	Metropolitan (*n* = 228)	Regional (*n* = 89)	*p*-Value
Mean overall survival, years (95% CI)	3.98 (3.70–4.25)	3.77 (3.44–4.09)	4.20 (3.76–4.63)	**0.019**
Overall survival rates (%)				
1-year	85.2%	84.6%	85.9%	
2-year	72.0%	69.1%	79.2%	
3-year	60.4%	54.2%	75.9%	
5-year	48.3%	44.1%	58.4%	
Recurrence,* frequency (%)				**0.014**
Yes	94 (56.0%)	76 (61.8%)	18 (40.0%)	
No	74 (44.0%)	47 (38.2%)	27 (60.0%)	
Follow-up time, years ± SD	2.46 ± 1.44	2.40 ± 1.47	2.60 ± 1.38	0.280

Bold values denote statistical significance at p < 0.05 level

*Sample < 317 indicates missing data.

### Quality of life measurements

The PCS and MCS are presented from baseline to 12 months in Table 5. Comparing groups, metropolitan and regional patients recorded mean baseline PCS of 47.3 (SD: 9.3) and 46.7 (SD: 10.6), respectively, and MCS of 47.4 (SD: 9.9) and 48.0 (SD: 11.6), respectively (*p* = 0.690). From pre-discharge to 12 months, QoL scores gradually increased to above baseline levels for both groups, except for the PCS for metropolitan patients [47.3 (SD: 9.3) versus 46.3 (SD: 10.4)]. The QoL scores between groups at similar time points were mostly comparable, with no statistically significant differences.

**Table 5:** Longitudinal quality of life outcomes at multiple time points

	Overall	Metropolitan	Regional	*p*-Value
Physical component score (PCS)				
Baseline*	47.1 ± 9.6 (*n* = 239)	47.3 ± 9.3 (*n* = 171)	46.7 ± 10.6 (*n*= 68)	0.678
Pre-discharge*	35.6 ± 8.7 (*n*= 233)	35.5 ± 8.6 (*n*= 168)	35.9 ± 9.0 (*n* = 65)	0.806
3 months*	44.0 ± 9.5 (*n* = 176)	44.1 ± 9.5 (*n* = 126)	43.8 ± 9.4 (*n* = 50)	0.863
6 months*	46.0 ± 9.8 (*n* = 155)	45.6 ± 9.8 (*n*= 110)	46.7 ± 9.8 (*n*= 45)	0.523
12 months*	47.3 ± 10.1 (*n* = 100)	46.3 ± 10.4 (*n* = 71)	49.6 ± 9.1 (*n* = 29)	0.142
Mental component score (MCS)				
Baseline*	47.6 ± 10.4 (*n* = 239)	47.4 ± 9.9 (*n* = 171)	48.0 ± 11.6 (*n* = 68)	0.690
Pre-discharge*	44.5 ± 11.5 (N = 233)	44.5 ± 11.3 (N = 168)	44.3 ± 12.2 (N = 65)	0.928
3 months*	47.6 ± 10.6 (*n* = 176)	47.6 ± 10.8 (*n*= 126)	47.5 ± 10.3 (*n* = 50)	0.934
6 months*	48.4 ± 9.6 (*n*= 155)	48.5 ± 9.6 (*n*= 110)	48.1 ± 10.5 (*n* = 45)	0.790
12 months*	48.4 ± 11.0 (*n* = 100)	48.0 ± 10.5 (*n* = 71)	49.5 ± 12.2 (*n* = 29)	0.554

Data presented as mean ± standard deviation

*Sample < 317 indicates missing data

## Discussion

In this study of patients undergoing CRS and HIPEC, most oncological parameters, surgical outcomes and QoL measurements were equally favourable in metropolitan and regional groups. These results are promising, considering that previous research had demonstrated delayed oncological diagnosis owing to limited healthcare access and lower health literacy in regional Australia [31, 32]. These challenges were further compounded by unique obstacles that regional patients face when seeking specialised surgical care, including psycho-social issues linked to separation, logistical issues related to travel, finding time off work and accommodation expenses [33, 34]. However, when examining oncological parameters in the current study, there were no statistical differences for PCI and CC score. This suggests that regional patients who were referred and treated at a specialist quaternary referral centre could expect to achieve oncological outcomes similar to those of metropolitan patients. Additionally, these promising results may also be a consequence of Australia’s focus towards centralising specialist surgical care [35].

The majority of surgical outcomes, including LOS and complication rates revealed no significant differences between the groups. Further analysis in the post-operative period revealed higher rates of recurrence for metropolitan patients. This was also reflected in the overall survival rate (Table 4), with longer survival for regional patients (Fig. 1). One systematic review by Ireland et al. [18], while not specifically focusing on CRS and HIPEC, reported inconsistent levels of evidence of survival disparities on the basis of geographical status for colorectal cancer in Australia. Other oncological studies have reported no significant geographical influence on predicting survival for colorectal cancer, attributing this to improvements in regional health infrastructure [36, 37].

In this study, there were no measurable advantages for metropolitan patients compared with regional patients treated by CRS and HIPEC in a peritoneal malignancy. To better understand the recurrence and survival patterns, several points of discussion warrant further review. Firstly, some regional patients received follow-up care from their referring surgeon, potentially leading to variations in follow-up protocols. Combined with the likelihood of limited experience of managing CRS and HIPEC patients, the detection of recurrence in regional patients might be delayed or diminished. Metropolitan patients were more likely to be followed-up with by the treating CRS and HIPEC surgeon. Additionally, follow-up protocols may have also been influenced by coronavirus disease 2019 (COVID-19) travel restrictions in 2020–2021, which limited movement and ability to attend diagnostic imaging or in-person follow-up appointments within New South Wales.

Secondly, another factor that may have impacted survival rate is lower health literacy in regional Australia [38], which is associated with poorer health outcomes and underutilisation of healthcare services [39]. Hence, the regional patients that were referred to our specialist surgical centre likely represented a selective subset of the regional population that was comparatively healthier, was more financially capable and possessed higher levels of health literacy. Collectively, these factors allowed for smoother navigation of the surgical referral pathway. Additionally, these findings emphasise the multi-faceted nature of health literacy disparities, where the literature highlights the considerable, but not entirely understood, influence of socio-demographic factors [38]. Therefore, the regional patients in this study were likely to be highly selective; this could be a contributing factor to the observed survival difference.

Nevertheless, the findings of this study indicate that regional patients may achieve equally favourable outcomes as metropolitan patients, particularly for pre-operative and intra-operative surgical outcomes and oncological parameters. However, as patients return to their primary residence in the post-operative period, they may encounter limited healthcare and support services [40], potentially affecting their post-operative QoL [41]. In our study, there were no statistically significant differences in QoL scores between metropolitan and regional patients up to 12 months post-operatively. When examining QoL by their components, all scores were higher than baseline except for metropolitan physical component score. This gradual rise above baseline may also reflect the multi-disciplinary approach afforded to CRS and HIPEC patients at a specialised quaternary referral centre. In addition to review by medical and surgical specialists, patients receive evaluations from surgical nursing staff, psychologists, physiotherapists and other allied health professionals at multiple stages of the perioperative period. Consequently, these services remain accessible to regional patients after discharge, potentially contributing to the similar QoL results observed.

### Strengths and limitations

Owing to the retrospective nature of this study, several limitations warrant further discussion. Firstly, only patients who were recommended for surgery and were referred to our specialised quaternary referral centre were considered for CRS and HIPEC, excluding those who were not referred but may have still benefited from surgery. This raises the possibility of selection bias and may have contributed to subtle differences between groups for oncological, surgical and QoL outcomes. Secondly, the single-centre methodology limits the generalisability of these findings to other healthcare facilities. However, given the scarcity of CRS and HIPEC research in Australia, these findings are considered significant.

The major strengths of this study were the high number of patients who consented for baseline analysis, standardised protocol of CRS and HIPEC, and comprehensive follow-up data from a reliable hospital database. Future studies are required to describe patient pathways where surgery was not recommended and to better understand referral pathways for regional and rural patients to centralised specialist surgical centres.

## Conclusions

The findings of this study indicate that geographical location of patients undergoing CRS and HIPEC at a high-volume quaternary centre had no discernible impact on oncological, surgical and QoL outcomes. For regional patients, their pre-operative and intra-operative surgical and oncological outcomes were similar to their metropolitan counterparts and reflect the trend towards centralisation of specialised oncological services. While there were differences in recurrence and survival, these findings may be a consequence of a number of factors, including non-standardised follow-up protocols, public health mandates during COVID-19 and varying levels of health literacy. Additionally, selection bias could have influenced these results since this study only included selective patients who were referred to our highly specialised centre and underwent surgical treatment. Therefore, further research should prioritise the characterisation of surgical referral pathways from regional and rural locations. Regardless of geographical residence, this study suggests that, when receiving CRS and HIPEC in experienced peritoneal malignancy centres, there are no measurable advantages for metropolitan patients compared with regional patients.

## Data Availability

The datasets generated during and/or analysed during the current study are available from the corresponding author on reasonable request.

## References

[1] Australian Institute of Health and Welfare (2022) Australia’s health 2022. Australian Institute of Health and Welfare: Canberra, https://www.aihw.gov.au/reports-data/australias-health

[2] Australian Institute of Health and Welfare (2022) Rural and remote health. Australian Institute of Health and Welfare: Canberra, https://www.aihw.gov.au/reports/rural-remote-australians/rural-and-remote-health

[3] Australia Standing Council on Health (2012) National strategic framework for rural and remote health/standing council on health. Rural and Regional Health Australia, http://nla.gov.au/nla.arc-149260

[4] Barclay L, Phillips A, Lyle D (2018) Rural and remote health research: does the investment match the need? Aust J Rural Health 26(2):74–79 29624788 10.1111/ajr.12429 PMC5947543

[5] Disler R, Glenister K, Wright J (2020) Rural chronic disease research patterns in the United Kingdom, United States, Canada, Australia and New Zealand: a systematic integrative review. BMC Public Health 20(1):770 32448173 10.1186/s12889-020-08912-1 PMC7247224

[6] Bosma NA et al. (2020) Characterizing urban-rural differences in colon cancer outcomes: a population-based analysis based on travel distance to cancer center. Am J Clin Oncol 43(7):531–535 32324599 10.1097/COC.0000000000000703

[7] Mullan L, Armstrong K, Job J (2023) Barriers and enablers to structured care delivery in Australian rural primary care. Aust J Rural Health 31(3):361–384 10.1111/ajr.12963 36639909

[8] Teutsch S, et al. (2023) Australian children living with rare diseases: health service use and barriers to accessing care. World J Pediatr, 19(7):701–709 10.1007/s12519-022-00675-6 PMC9848027 36653598

[9] O’Sullivan B (2022) Challenges and innovations in access to community-based rural primary care services during the COVID-19 pandemic in Australia. Int J Health Plann Manage 37(Suppl 1):115–128 36443892 10.1002/hpm.3598 PMC9878203

[10] Hussain R et al. (2015) The fly-in fly-out and drive-in drive-out model of health care service provision for rural and remote Australia: benefits and disadvantages. Rural Remote Health 15(3):357–363 26190237

[11] Shukla N et al. (2020) A review of models used for investigating barriers to healthcare access in Australia. Int J Environ Res Public Health 17(11):4087 32521710 10.3390/ijerph17114087 PMC7312585

[12] Crawford-Williams F et al. (2018) Cancer care in regional Australia from the health professional’s perspective. Support Care Cancer 26(10):3507–3515 29696425 10.1007/s00520-018-4218-x

[13] Foster JM et al. (2019) Morbidity and mortality rates following cytoreductive surgery combined with hyperthermic intraperitoneal chemotherapy compared with other high-risk surgical oncology procedures. JAMA Netw Open 2(1):e186847 30646202 10.1001/jamanetworkopen.2018.6847 PMC6484874

[14] Anwar A, Kasi A (2022) Peritoneal cancer. StatPearls. StatPearls Publishing 32965809

[15] Lungoci C et al. (2016) Multimodality treatment strategies have changed prognosis of peritoneal metastases. World J Gastrointest Oncol 8(1):67–82 26798438 10.4251/wjgo.v8.i1.67 PMC4714147

[16] Mehta SS, Bhatt A, Glehen O (2016) Cytoreductive surgery and peritonectomy procedures. Indian J Surg Oncol 7(2):139–151 27065704 10.1007/s13193-016-0505-5 PMC4818624

[17] Mohamed F et al. (2011) A new standard of care for the management of peritoneal surface malignancy. Curr Oncol 18(2):e84-96 21505593 10.3747/co.v18i2.663 PMC3070715

[18] Ireland MJ et al. (2017) A systematic review of geographical differences in management and outcomes for colorectal cancer in Australia. BMC Cancer 17(1):95 28152983 10.1186/s12885-017-3067-1 PMC5290650

[19] Cuschieri S (2019) The STROBE guidelines. Saudi J Anaesth 13(Suppl 1):S31-s34 30930717 10.4103/sja.SJA_543_18 PMC6398292

[20] Australian Bureau of Statistics (2021) Australian statistical geography standard (ASGS) Edition 3, https://www.abs.gov.au/statistics/standards/australian-statistical-geography-standard-asgs-edition-3/latest-release

[21] Jacquet P, Sugarbaker PH (1996) Clinical research methodologies in diagnosis and staging of patients with peritoneal carcinomatosis. Cancer Treat Res 82:359–374 8849962 10.1007/978-1-4613-1247-5_23

[22] Wong JSM et al. (2022) Implications of peritoneal cancer index distribution on patients undergoing cytoreductive surgery and hyperthermic intraperitoneal chemotherapy. Pleura Peritoneum 7(2):95–102 35812008 10.1515/pp-2021-0150 PMC9166179

[23] Wu HT et al. (2016) Cytoreductive surgery plus hyperthermic intraperitoneal chemotherapy with lobaplatin and docetaxel to treat synchronous peritoneal carcinomatosis from gastric cancer: results from a Chinese center. Euro J Surg Oncol (EJSO) 42(7):1024–1034 10.1016/j.ejso.2016.04.053 27179924

[24] Sinukumar S et al. (2019) Analysis of clinical outcomes of pseudomyxoma peritonei from appendicular origin following cytoreductive surgery and hyperthermic intraperitoneal chemotherapy-a retrospective study from INDEPSO. Indian J Surg Oncol 10(Suppl 1):65–70 30886496 10.1007/s13193-018-00870-w PMC6397130

[25] Dindo D, Demartines N, Clavien PA (2004) Classification of surgical complications: a new proposal with evaluation in a cohort of 6336 patients and results of a survey. Ann Surg 240(2):205–213 15273542 10.1097/01.sla.0000133083.54934.ae PMC1360123

[26] Dindo D (2014) The Clavien–Dindo classification of surgical complications. Treatment of postoperative complications after digestive surgery 250(2):187–196 10.1097/SLA.0b013e3181b13ca2 19638912

[27] Ware JE Jr, Sherbourne CD (1992) The MOS 36-item short-form health survey (SF-36). I. Conceptual framework and item selection. Med Care 30(6):473–83 1593914

[28] Steffens D et al. (2023) Surgical, survival and quality of life outcomes in over 1000 pelvic exenterations: lessons learned from a large Australian case series. ANZ J Surg 93(5):1232–1241 36869215 10.1111/ans.18356

[29] Steffens D et al. (2020) Quality of life after cytoreductive surgery and hyperthermic intraperitoneal chemotherapy: early results from a prospective cohort study of 115 patients. Ann Surg Oncol 27(10):3986–3994 32285283 10.1245/s10434-020-08443-4

[30] Ware J, M Kosinski, S Keller (1994) SF-36 physical and mental health summary scales. A user’s manual. Boston, MA.

[31] Emery JD et al. (2013) Diagnosing cancer in the bush: a mixed-methods study of symptom appraisal and help-seeking behaviour in people with cancer from rural Western Australia. Fam Pract 30(3):294–301 23363540 10.1093/fampra/cms087

[32] Bergin RJ et al. (2018) Rural-urban disparities in time to diagnosis and treatment for colorectal and breast cancer. Cancer Epidemiol Biomark Prev 27(9):1036–1046 10.1158/1055-9965.EPI-18-0210 29987098

[33] Jazdarehee A, Parajulee A, Kornelsen J (2021) The experiences of rural British Columbians accessing surgical and obstetrical care. Patient Exp J 8(1):126–134

[34] Stewart GD, Long G, Tulloh BR (2006) Surgical service centralisation in Australia versus choice and quality of life for rural patients. Med J Aust 185(3):162–163 16893360 10.5694/j.1326-5377.2006.tb00507.x

[35] Carter J, Pather S, Nascimento M (2018) Current status of ovarian cancer surgical management. Argument for centralisation of care in Australia. Aust N Z J Obstet Gynaecol 58(4):474–477 29851066 10.1111/ajo.12832

[36] Singla A et al. (2014) Rural populations have equal surgical and survival outcomes in metastatic colorectal cancer. Aust J Rural Health 22(5):249–256 25303417 10.1111/ajr.12133

[37] MacVicar E et al. (2020) Analysing the impact of living in a rural setting on the presentation and outcome of colorectal cancer. A prospective single centre observational study. Surg 18(6):354–359 10.1016/j.surge.2020.02.001 32184069

[38] Aljassim N, Ostini R (2020) Health literacy in rural and urban populations: a systematic review. Patient Educ Couns 103(10):2142–2154 32601042 10.1016/j.pec.2020.06.007

[39] Berkman ND et al. (2011) Low health literacy and health outcomes: an updated systematic review. Ann Intern Med 155(2):97–107 21768583 10.7326/0003-4819-155-2-201107190-00005

[40] Goodwin BC et al. (2023) What are the post-treatment information needs of rural cancer survivors in Australia? A systematic literature review. Psychooncology (Chichester, England) 32(7):1001–1012 10.1002/pon.6169 37248643

[41] Gunn KM et al. (2021) Improving survivors’ quality of life post-treatment: the perspectives of rural Australian cancer survivors and their carers. Cancers (Basel) 13(7):1600 33808464 10.3390/cancers13071600 PMC8037228

